# Quasi-BIC based all-dielectric metasurfaces for ultra-sensitive refractive index and temperature sensing

**DOI:** 10.1038/s41598-023-48051-2

**Published:** 2023-11-23

**Authors:** Seyedeh Bita Saadatmand, Vahid Ahmadi, Seyedeh Mehri Hamidi

**Affiliations:** 1 Faculty of Electrical and Computer Engineering, Tarbiat Modares University, Tehran, Iran; 2 Magneto-Plasmonic Lab, Laser and Plasma Research Institute, Shahid Beheshti University, Tehran, Iran

**Keywords:** Nanoscience and technology, Optics and photonics

## Abstract

In this paper, an all-dielectric metasurface that measures refractive index and temperature using silicon disks is presented. It can simultaneously produce three resonances excited by a magnetic toroidal dipole, magnetic toroidal quadrupole, and electric toroidal dipole, in the THz region. Asymmetric structures are used to generate two quasi-bound states in the continuum (BIC) resonances with ultra-high-quality factors driven by magnetic and electric toroidal dipoles. We numerically study and show the dominant electromagnetic excitations in the three resonances through near-field analysis and cartesian multipole decomposition. The effects of geometric parameters, scaling properties, polarization angles, incident angles, and silicon losses are also investigated. The proposed metasurface is an excellent candidate for sensing due to the extremely high-quality factor of the quasi-BICs. The results demonstrate that the sensitivities for liquid and gas detection are S_l_ = 569.1 GHz/RIU and S_g_ = 529 GHz/RIU for magnetic toroidal dipole, and S_l_ = 532 GHz/RIU and S_g_ = 498.3 GHz/RIU for electric toroidal dipole, respectively. Furthermore, the sensitivity for temperature monitoring can reach up to 20.24 nm/°C. This work presents a valuable reference for developing applications in the THz region such as optical modulators, multi-channel biochemical sensing, and optical switches.

## Introduction

Recently, the THz band, covering a region between 100 GHz to 30 THz, has garnered significant interest due to its ability to address sensing challenges complementarity with other spectrum regions^1–3^. However, increasing the sensitivity of THz sensors is necessary to fulfill the requirements of industrial and clinical applications. Metallic and all-dielectric metasurfaces, artificial surfaces made of meta-atoms, can sustain Mie resonances and have the potential to be used for the implementation of ultrasensitive sensors required for THz sensing where we need to improve the interaction with the surrounding medium^4^. Numerous metasurface-based techniques have been developed to improve the performance of THz sensors^5–8^. A comprehensive comparison by A. Ahmadivand et al. provides insights into various THz metasensors, highlighting that THz plasmonic metasensors based on toroidal technology are well-suited for modern clinical and laboratory applications. These metasensors are promising in detecting and recognizing biomarker proteins associated with diseases such as Alzheimer's and Parkinson's, even at ultra-low densities. The investigation also studies the sensing performance in terms of sensitivity, limit of detection, figure of merit, selectivity, repeatability, and the detection of various materials^9^. In another study, the advancements and operating principles of various types of metasensors, encompassing hyperbolic, Fano-resonant, chiral, surface lattice resonance, Tamm modes, magnetic, toroidal, and quasi-bound states in the continuum are discussed. These metasensors offer diverse applications across multiple scientific and medical domains. They can be utilized for cost-effective and efficient diagnosis of infectious diseases and non-invasive live-cell molecular imaging even at extremely low concentrations^10^.

To recognize a small frequency shift caused by a weak environmental change, a high-quality factor (Q-factor) resonance is typically needed for sensing applications. Additionally, the resonance's intensity should be sufficient to allow for easy detection in a noisy environment^11^. The bound state in the continuum (BIC) as confined states located within the continuum is an especially effective technique for achieving high Q resonance^12^. Such states allow the energy to be perfectly contained within the system without radiation^13,14^. A BIC can therefore be viewed as a resonance with an infinite Q-factor or zero linewidth. Theoretically, BICs are infinite-lifetime dark modes that are invisible in far-field measurements but observable in near-field experiments^15^. However, because of the finite size of structures, material absorption, and other disturbances, BICs frequently collapse to Fano resonances, exhibiting finite radiative Q values, and are denoted as quasi-BICs (q-BICs)^16–18^. The two primary BIC classes in metasurfaces are Friedrich-Wintgen and symmetry-protected BICs (sp-BICs)^19,20^. The coupling coefficient may disappear if the mode spatial symmetry is irreconcilable with the symmetry of the outgoing waves^21^. The ideal sp-BIC can be converted into a q-BIC by opening a radiation channel through perturbing structural symmetry or oblique incidence, whereas the Friedrich-Wintgen BIC is produced by destructive interference of the eigenmodes, which necessitates exact adjusting of the structure parameters^22–24^. Despite the rapid development of high Q resonances of BIC metasurfaces in the optical band, there have been some challenges in the THz regime.

While the far-field analysis of all-dielectric metasurface q-BICs shows Fano resonances, their near-field electromagnetic patterns differ significantly. The analysis of the q-BIC field distributions can be performed through electromagnetic multipole decomposition. Accordingly, BICs can be categorized based on their predominant electromagnetic multipolar characteristics, such as magnetic dipole (MD), electric dipole (ED), toroidal dipole (TD), etc^22,25,26^. The TD is a unique type of localized electromagnetic excitation that makes it different from the magnetic and electric dipoles. While the electric dipole can be visualized as a pair of opposing charges and the magnetic dipole as a circular current loop, the TD involves currents flowing specifically on the surface of a torus, which is a donut-shaped structure. Toroidal dipoles have a significant impact on the fundamental characteristics of matter, including dispersion, absorption, and optical activity^27,28^. TDs have been extensively investigated in various all-dielectric and plasmonic bulk meta platforms, spanning from infrared to microwave frequencies. They can be used in applications such as perfect absorption and permittivity sensing. However, challenges related to fabrication procedures, integration with planar devices, and losses have led researchers to focus on the development of toroidal metastructures based on flatland optics^29^. In flatland optics, researchers have focused on creating quasi-infinite metasurfaces made up of periodic arrays of scatterers or optical thin films. The researchers have extensively worked on generating TDs within an artificial medium consisting of engineered planar metallic and dielectric unit cells. The advantages of this approach include straightforward modeling and numerical calculations, cost-effective and easy fabrication, and reduced radiative losses. Consequently, artificial structures based on flatland optics attracted significant attention from researchers in this field^29^. The magnetic toroidal dipole (MTD) and the electric toroidal dipole (ETD) are two different categories of TDs, produced by magnetic dipoles aligned head-to-tail to create a ring and by a closed loop of electric dipoles, respectively^30^. The prior researches have mainly concentrated on studying the application and properties of the MTD-BIC. However, the ETD-BIC receives less attention and analysis, whereas a wide range of applications can be developed by identifying their particular response mechanisms, and classifying them in the spectrum^31–33^.

In this work, the toroidal dipole modes (MTD and ETD) are investigated in a novel all-dielectric metasurface. We follow the structure presented in Ref.^34^. The physical mechanism of the q-BIC modes is analyzed using cartesian multipolar decomposition and electromagnetic near-field distributions, which show that they are driven by TD. The TD resonances can be collapsed from sp-BIC to q-BIC with an extremely high Q-factor of $5.1 \times 10^{6}$ using the asymmetric parameter. We next investigate the effects of the geometric parameters, scaling properties, polarization angles, incident angles, silicon losses, and temperature on the characteristics of the suggested metasurface. The results show that the proposed structure's sensitivity can accede up to 532 GHz/RIU for liquid detection and 498.3 GHz/RIU for gas detection. This corresponds to a figure of merit (FoM) of 5,320,000 RIU^-1^, and 4,983,000 RIU^−1^, respectively, which, according to the best of our knowledge, is the highest reported value in the THz region. Additionally, the corresponding temperature sensitivity can reach 20.24 nm/°C. This sensitivity is enhanced compared to that of previously studied sensors based on all-dielectric metasurface in the THz region. Our study offers a novel perspective on toroidal moment excitation and development.

## Structure design and methods

**Figure 1:**
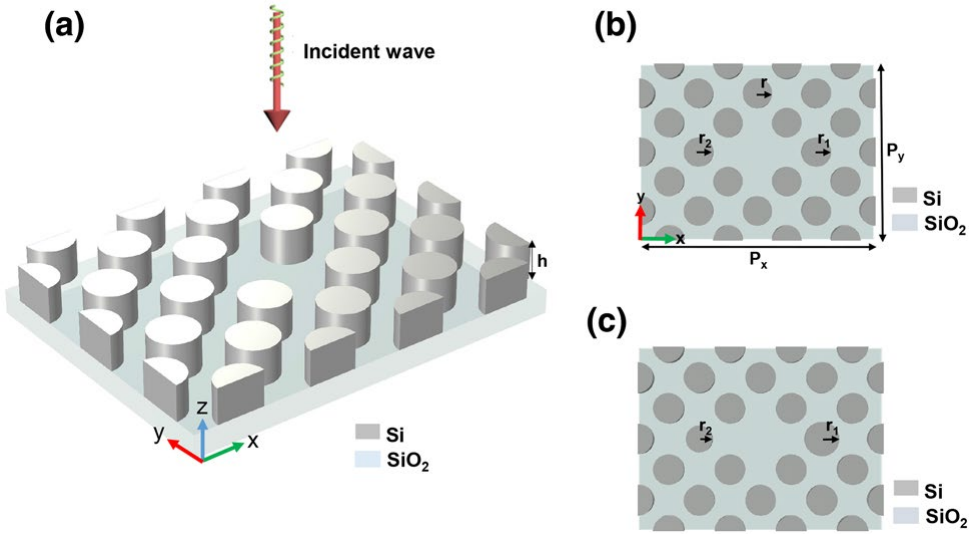
(**a**) Schematic of the proposed metastructure, (**b**) Symmetric, and (**c**) Asymmetric unit cell with the geometric parameters. *P*_*x*_ and *P*_*y*_ are equal to 640 µm and 480 µm, respectively. The silicon disks have a thickness of *h* = 60 µm and a radius of *r* = 40 µm.

Figure 1 depicts the proposed multiple Fano resonance device based on periodic disks. The periods along the x (*P*_*x*_) and y axes (*P*_*y*_) are equal to 640 µm and 480 µm, respectively. Silicon disks on a SiO_2_ substrate compose the metasurface unit cell. The silicon disks have a thickness of *h* = 60 µm and a radius of *r* = 40 µm. The asymmetry parameter is defined by $a = r - (r_{1} - r_{2} )$, where *r*_*1*_ and *r*_*2*_ are the radii of the two disturbed disks as shown in Fig. 1b. Figure 1c shows that when $r_{1} \ne r_{2} \ne r$, the metasurface is changed to an asymmetric structure. The propagation direction of a plane wave is along the z-axis, and its electric field is polarized in the x-axis. Numerical simulations are carried out through the implementation of a three-dimensional finite-element approach (see Section S1). The optical constants of Si and SiO_2_ are extracted from Ref.^35^. The simulation boundary conditions are a perfectly matched layer in the z-direction and periodic in the x and y-directions. The feasibility of experimental fabrication is presented in Section S2.

## Results and discussion

**Figure 2:**
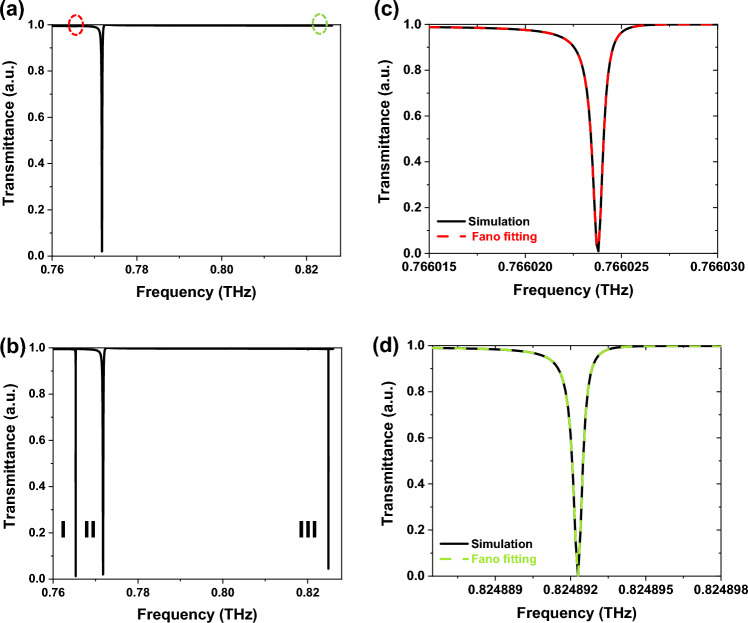
Transmittance curves with an asymmetric parameter (**a**) *α* = 0 µm, and (**b**) *α* = 2 µm. Three modes are shown by modes I, II, and III, respectively. (**c**,**d**) Fano fitting of mode I and mode III at *α* = 2 µm. The simulation results are shown in solid curves, while the results of the Fano fitting are shown in dashed curves.

The metasurface in-plane symmetry is disrupted when $r_{1} \ne r_{2} \ne r$, opening a new radiation pathway for the incident beam to leak into zero-order channel, which is the method by which sp-BIC transforms into q-BIC. Transmittance curves of symmetry and asymmetry structures are shown in Fig. 2a and b, respectively. When *α* = 0 µm a single Fano resonance (mode II) is observed, and when *α* $\ne$ 0 µm three Fano resonances (modes I, II, and III) are appeared. Here, mode II corresponds to the Mie resonance of symmetry structure and can always be seen and has nearly the same resonance frequency and extinction ratio because of the constant structure effective refractive index. However, modes I, and III are the BIC-related modes that are induced by breaking the symmetry. When $r_{1} = r_{2} = r$, modes I and III vanish, indicating that the radiation channel is closed and the bound state does not lose energy to the continuum, so, the radiative Q-factor approaches infinity, as identified by the circles in Fig. 2 (a). Therefore, by breaking the symmetry, two completely different Fano resonances are created by the interference between the continuous radiation and the discrete bound state supported by the structure. To calculate the Q-factor of BIC modes using ${\text{Q}} = \omega_{0} /2\gamma$, the conventional Fano formula is utilized^36^:

1 $$ T_{{{\text{Fano}}}} (\omega ) = \left| {a_{1} + ja_{2} + \frac{b}{{\omega - \omega_{0} + j\gamma }}} \right|^{2} , $$

where *ω*_*0*_ is the resonant frequency, *a*_*1*_, *a*_*2*_, and *b* are the constants, and *γ* is the total rate of damping and characterizes the Q-factor of the BICs. The fitting transmittance curves for modes I and III at *α* = 2 µm are shown in Fig. 2c and d, respectively. The resonance frequency of mode I is 0.7660238 THz with a Q-factor of $9 \times 10^{5}$, and mode III is located at 0.8248923 THz and has a Q-factor of $5.1 \times 10^{6}$ at *α* = 2 μm. These results validate the simulation results, which are represented by the black solid lines in Fig. 2c and d, respectively.

As can be observed in Fig. 2b, besides the high Q-factor of BIC modes, the transmittance spectrum of the proposed structure is free of unnecessary and clutter modes that could interfere with the resonant states in the specified wavelength range. In addition, in the proposed structure, the spectral contrast ratio and modulation depth of all modes in the wavelength range of interest reach about 100%, which are defined as [(T_on_ − T_off_)]/[(T_on_ + T_off_)] × 100% and [(T_on_ − T_off_)/T_on_] × 100%, where T_off_ and T_on_ refer to the minimum and maximum transmittance, respectively^36^. Accordingly, thanks to its extremely high modulation depths and spectral contrast ratio, the proposed metasurface successfully prevents the impact of noise on the accuracy of detection.

Furthermore, the optical resonance is characterized by its effective mode volume, a significant parameter that describes how electromagnetic energy is distributed within the structure. Specifically, it is determined by the ratio of the total energy of the q-BICs inside the structure to the maximum energy density. This distribution of energy density in three-dimensional space can be represented as Ref.^10^:

2 $$ V_{eff} = \frac{{\int_{V} {\varepsilon (r)\left| {E(r)} \right|^{2} dV} }}{{\max \left\{ {\varepsilon (r)\left| {E(r)} \right|^{2} } \right\}}}, $$

where *ε(r)* represents the permittivity, *V* is the total calculation volume, and *E(r)* represents the distribution of the electric field within the structure. Analyzing Eq. (2) reveals that when the intensity of the incident light remains constant, a smaller effective mode volume results in a larger Q/V_eff_ value. Consequently, this concentration of electric field energy within the structure facilitates a strong interaction between light and matter. For the q-BIC resonances, the effective mode volumes are calculated as: 1.36 × 10^–11^ m^3^ for mode I and 2.92 × 10^–10^ m^3^ for mode III. Because photons are confined to the structure, the mode volumes are significantly reduced. This localization of the incident field also leads to enhanced effects in the near field. The proposed metasurface exhibits a high Q/V_eff_ value, which makes it promising for the sensing application.

Another important factor to consider is the dephasing time of resonances, which is a critical parameter determined by the narrowness of the resonance. The dephasing time can be calculated using *T* = *2ℏ/δ*, where *ℏ* represents the reduced Planck's constant and *δ* is the homogeneous linewidth of the resonance^37^. The estimated dephasing times for mode I and mode III are 0.3 µs and 1.9 µs, respectively.

**Figure 3:**
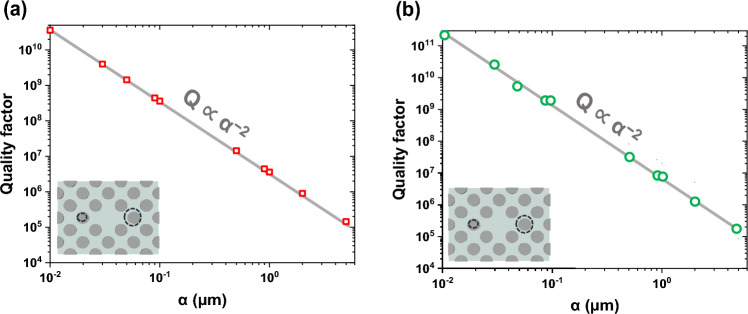
Quality factors of the (**a**) mode I, and (**b**) mode III for different asymmetry parameters. The red and green points are obtained by the finite element method, and the black line is fitted to show that the Q-factor and α of modes I and III follow the inverse quadratic law as Q ∝ α ^−2^.

Figure 3a and b demonstrate that the Q-factor and *α* of modes I and III follow the inverse quadratic law as Q ∝ *α*^−2^. So, modes I and III originate from sp-BIC^38^. As a result, it is possible to manipulate the Q value of these modes by modifying the asymmetric metasurface characteristics. The results show that in the proposed structure, a high Q-factor is possible with the asymmetry parameter of the micrometer scale. This is due to the fact that coupling between meta-atoms in the proposed metastructure leads to light confinement which increases the interaction between light and matter. While achieving such a quality factor in other structures is obtained with a smaller asymmetry parameter^39–41^. Because of the limitations of existing fabrication process technology, it is very hard to produce such small asymmetric changes in structures. However, for the proposed structure the fabrication process is more feasible.

**Figure 4:**
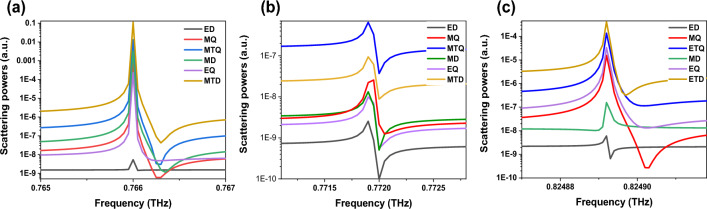
The scattering powers of multipole decomposition for (**a**) mode I, (**b**) mode II, and (**c**) mode III. MTD is dominant in mode I, MTQ is dominant in mode II, and ETD has the main contributions in mode III.

To better study the characteristics of Fano modes, the scattering power for various kinds of multipole excitations is computed using the cartesian multipole decomposition method. The displacement current density *j(r)* is integrated over the unit cell to provide a thorough description of the near-field and far-field response of electromagnetic sources. The total far-field scattered power for all multipoles is then determined for a given frequency. The eight most significant multipoles are considered, as defined by ED:$P = \frac{1}{i\omega }\int {jd^{3} r}$, MD:$M = \frac{1}{2c}\int {(r \times j)} d^{3} r$, MTD:$T = \frac{1}{10c}\int {\left[ {(r.j)r - 2r^{2} j} \right]} d^{3} r$, ETD:$T^{e} = \frac{{\omega^{2} }}{{20c^{2} }}\int {\left[ {(r \times j)_{\alpha } r^{2} } \right]} d^{3} r$, electric quadrupole (EQ): $Q_{\alpha \beta }^{e} = \frac{1}{2i\omega }\int {\left[ {r_{\alpha } j_{\beta } + r_{\beta } j_{\alpha } - \frac{2}{3}(r.j)\delta_{\alpha ,\beta } } \right]} d^{3} r$, magnetic quadrupole (MQ): $Q_{\alpha \beta }^{m} = \frac{1}{3c}\int {\left[ {(r \times j)_{\alpha } r_{\beta } + (r \times j)_{\beta } r_{\alpha } } \right]} d^{3} r$, electric toroidal quadrupole (ETQ): $Q_{\alpha \beta }^{ET} = \frac{i\omega }{{42}}\int {r^{2} \left[ {r_{\alpha } (r \times j)_{\beta } + r_{\beta } (r \times j)_{\alpha } } \right]} d^{3} r$, and magnetic toroidal quadrupole (MTQ): $Q_{\alpha \beta }^{MT} = \frac{1}{28c}\int {\left[ {4r_{\alpha } r_{\beta } (r.j) - 5r^{2} (r_{\alpha } j_{\beta } + r_{\beta } j_{\alpha } ) + 2r^{2} \delta_{\alpha \beta } (r.j)} \right]} d^{3} r$, where *ω* is the angular frequency, *r*, and *c* are the position vectors and light speed, respectively, *δ* is the Dirac delta function, and subscripts *α*, *β* = x, y, z^42,43^. The scattered power of each multipole moment can be calculated from Refs.^44,45^. It should be pointed out that because of their insignificant contribution, multipole expansion at higher orders is not considered in the calculations. The multipole decompositions of the structure are shown in Fig. 4. Figure 4a shows that for mode I, MTD is dominant, with a partial contribution of the MTQ, MD, and MQ, and the other multipoles are dramatically suppressed in this case. However, in Fig. 4b, MTQ is dominant and the contribution of others is negligible. In Fig. 4c ETD has the main contributions in mode III. Notes, ETD in this mode is approximately 1 order stronger than EQ and MQ, 3 orders larger than MD, and around 5 orders stronger than ED.

**Figure 5:**
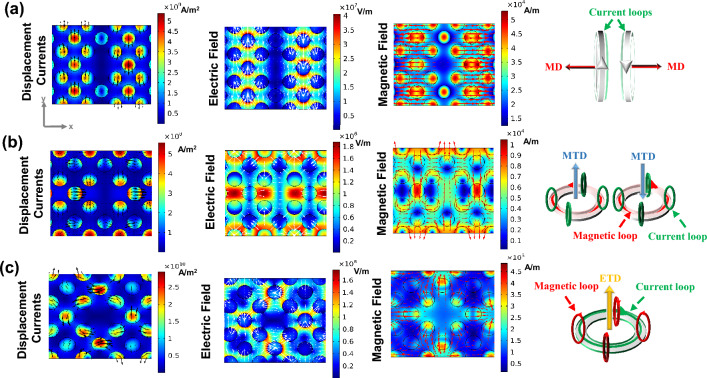
Cross-sectional patterns of displacement currents (black arrows), electric (white arrows), and magnetic (red arrows) fields in the x–y plane for the asymmetric structure for (**a**) mode I, (**b**) mode II, and (**c**) mode III.

**Figure 6:**
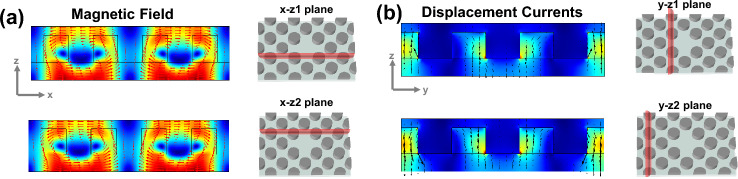
(**a**) The magnetic field patterns in x-z1 and x-z2 planes, and (**b**) The displacement currents in y-z1 and y-z2 planes. The red and black arrows demonstrate the flow direction of the magnetic field and displacement currents, respectively.

Additionally, the near-field patterns of the asymmetric unit cell in the x–y plane close to the three resonances as well as a schematic of the associated electromagnetic sources are shown in Fig. 5 to provide an intuitive explanation of the various electromagnetic excitations that correspond to each resonance. The displacement currents in mode I act circularly, turning around the center of meta-atoms in the y–z plane and generating magnetic moments oriented parallel to the x-axis (see the first row in Fig. 5). The multipole decomposition result in Fig. 4a, which indicates that the strongest mode should be MTD at mode I, seems to contradict this conclusion. Indeed, the different multipole excitation directions can account for this contradiction. According to the near-field distributions in Fig. 5a, the MD and MQ responses are dominated by the x component. However, for MTD response, the y component is strongest. As a result, in Fig. 6a, we show the magnetic field patterns of the structure in mode I in two x–z planes (x-z1 plane and x-z2 plane). The magnetic fields generated by opposite-direction displacement currents are depicted by the vortex arrows. This exhibits MTD characteristics along the y-axis.

The vortex arrows in Fig. 5b show that in each half of the x–y plane, two magnetic moment loops are circling in opposition to one another. As shown in Fig. 6b, a ring of the clockwise and anticlockwise rotating displacement currents occurs in the y–z plane; and is recognized as an MTD along the z-axes. The two MTD moments exhibit opposing directions along the z-axis. Figure 5c displays a clear vortex of displacement currents passing through disks in mode III, whereas the torus meridians are the direction of the magnetic fields, which makes it simple to identify an ETD. The inter-ETD moment along the z-axis is also displayed by clockwise circular displacement currents in the neighboring unit cells. As a result, we can conclude that the intra- and inter-ETD moments are both responsible for the ETD resonance excitation.

**Figure 7:**
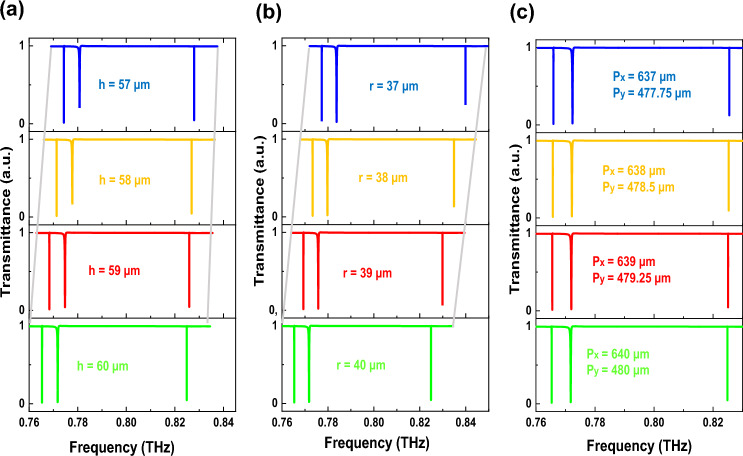
Transmittance curves of the proposed structure (*α* = 2 μm) with various (**a**) heights, (**b**) radius, and (**c**) periods. Adjusting the geometrical parameters leads to tuning the resonance frequency without noticeably affecting their Q-factor.

We calculate the response of the proposed metasurface with various geometric characteristics to analyze the relationship between transmittance spectra and geometric parameters. We set *α* = 2 µm and except for the variable parameter indicated in each curve, all other geometrical values are identical to those in Fig. 1. Figure 7a shows that as the height (*h*) increases from 57 to 60 µm, all resonances exhibit a redshift but their linewidths hardly change and the shift for ETD is the least value of the three resonances. Because in the ETD mode, the displacement current is in-plane, but in the other two modes, the current is out-of-plane, therefore the change in the height of the structure has a lower impact on ETD. According to Fig. 7b, all resonances shift to the red when the radius (*r*) is increased from 37 to 40 µm, and the ETD shift is more significant. Figure 7c shows that with increasing the period along the x-axis (*P*_*x*_) from 637 to 640 µm, because of the structure's higher effective refractive index, there are little redshifts for all the resonances. To discuss the redshift of the modes we can refer to the dielectric resonator's Mie resonance frequency explained as Ref.^46^:

3 $$ f = \frac{{\theta c^{2} }}{{2\pi V(x,y,z)\sqrt {\mu \varepsilon } }}. $$

The function *V (x, y, z)* is related to the size of the dielectric resonator, while *μ* and *ε* represent its permeability and permittivity, respectively. In addition, *θ* is a constant value that applies to a specific resonance. The rise in height, radius, and period can cause the device volume *V (x, y, z)* to increase, as a result, the resonant frequency redshifts. Therefore, the shift in resonance frequencies caused by the aforementioned parameters is consistent with the Mie theory. Notably, all modes exhibit a narrow linewidth even when the structural parameters change. This characteristic indicates that the proposed structure remains unaffected by fabrication tolerances, making it highly advantageous for practical applications (see section S3).

**Figure 8:**
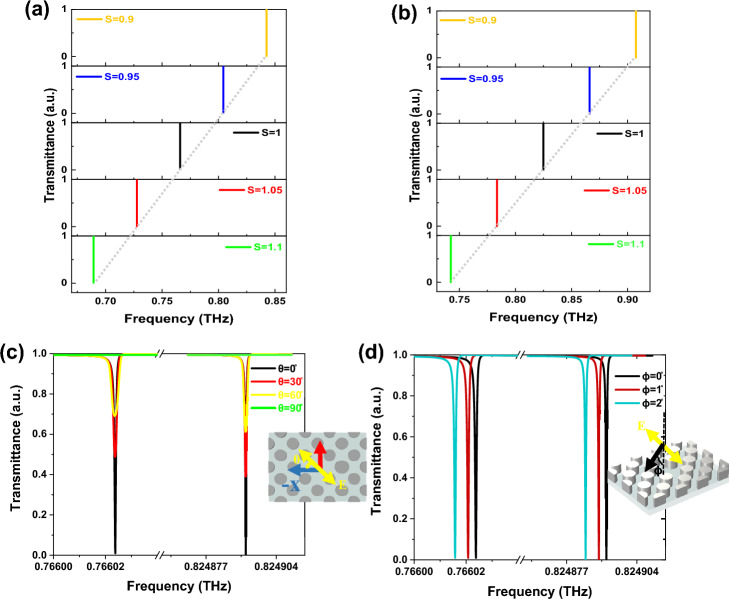
Transmittance curves (**a**) under different scalable factor S for mode I, and (**b**) mode III. The q-BICs exhibit linear redshifts as the scalable factor slowly increases, whereas their linewidth is approximately unchanged, indicating that S can be changed to tune the resonance frequencies. (**c**) Transmittance curves with different incident wave polarization directions (θ = 0 to 90°). As the θ rises from 0 to 90 degrees, the transmittance dips of modes I and III slowly broaden with no wavelength shifts, and (**d**) Transmittance curves with different incident wave angles (ɸ = 0 to 2°). The polarization angle and incident angle directions are shown in the insets of (**c**) and (**d**). The q-BIC resonances exhibit a redshift when the incident angle ranges from 0° to 2°

Moreover, it is desirable to examine the adjustable characteristics without changing the asymmetry parameter, so that the proposed metasurface may be employed in various frequency ranges to satisfy related demands; because Maxwell's equations in the dielectric configuration have scalability^47^. Therefore, we employ a scalability factor (S), and the geometrical parameters (*P*_*x*_, *P*_*y*_, *r*, and *h*) are multiplied by S. The results are demonstrated in Fig. 8a and b. The q-BICs exhibit linear redshifts as the scalable factor slowly increases, whereas their linewidth is approximately unchanged, indicating that S can be changed to tune the resonance frequencies.

The results of changes in the polarization direction of the incoming beam are also discussed. The transmittance curves of q-BIC modes with various polarization directions are shown in Fig. 8c, where it is possible to adjust the transmittance intensity of the Fano modes. The angle between the x direction and the orientation of the incident electric field is known as the polarization angle (θ), and it is depicted in the inset of Fig. 8c. As the θ rises from 0 to 90 degrees, the transmittance dips of modes I and III slowly broaden with no wavelength shifts. The resulting modulation depth of the Fano curves approaches almost 100% and zero at the angle of 0° and 90°, respectively. It shows that these sp-BIC modes are polarization-sensitive and appear at a particular incoming polarization angle. The suggested metasurface can also be utilized to develop highly efficient photonic switches, because of its huge Q value, extremely high spectrum contrast, and modulation depth. This device can perform more effectively than the plasmonic optical switches^48^. The impact of incident angles (ɸ) variation on the q-BIC resonances is shown in Fig. 8d. According to this figure, the q-BIC resonances exhibit a redshift when the incident angle ranges from 0° to 2°. Moreover, the magnitude of the redshift increases with the angle, while the linewidth remains relatively constant. Importantly, despite changes in the incident angle, the q-BIC resonances do not disappear, suggesting that the structure demonstrates a certain degree of tolerance towards angle deviations.

**Figure 9:**
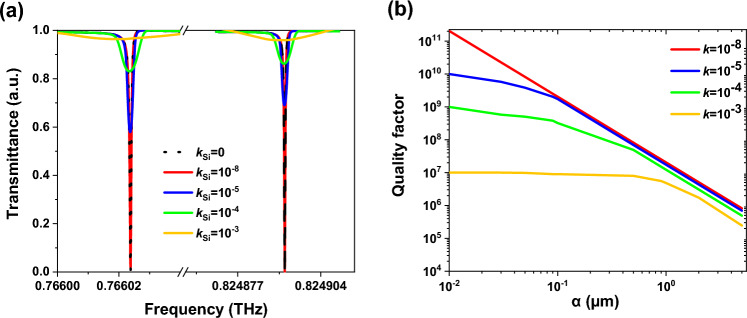
(**a**) Transmittance spectra of the q-BIC resonances for various values of silicon losses for *α* = 2 µm, (**b**) Dependence of the Q value of the mode III on silicon losses. The transmittance spectrum is found to be nearly unaffected for *k* ≤ 10^−8^. However, further increasing the *k* values, progressively dampens the resonance and degrades the Q-factor due to the Si absorption effect.

In practice, we don’t have real BIC, because the losses are inevitable factors that appear in metasurface manufacturing. Silicon has extremely low absorption losses in the THz region, however, surface roughness produced during the real fabrication process as well as absorption and scattering losses caused by defects are the main concern in the design of the metasurface. These losses are considered by adding the imaginary component of the silicon refractive index (*k*) (i.e., the extinction coefficient) as *n*_Si_ = *n*-*jk*. Figure 9a shows that for *α* = 2 µm, the transmittance spectrum is found to be nearly unaffected for *k* $\le$ 10^−8^. Therefore, in practical application, where *k* $\le$ 10^−8^ there are no obvious effects of the Si loss in the linewidth and Q-factor. However, further increasing the *k* values, progressively dampens the resonance and degrades the Q-factor due to the Si absorption effect. Figure 9b reports that when *k* = 10^–8^, the Q-factor drops as *α* rises, while when *k* = 10^–3^, the Q-factor remains almost constant for a wide range of *α*. When the asymmetry parameter is small, the field confined in the Si structure is stronger, so, the higher losses lead to much broadening in the resonance spectrum and consequently reduce the quality factor.

**Figure 10:**
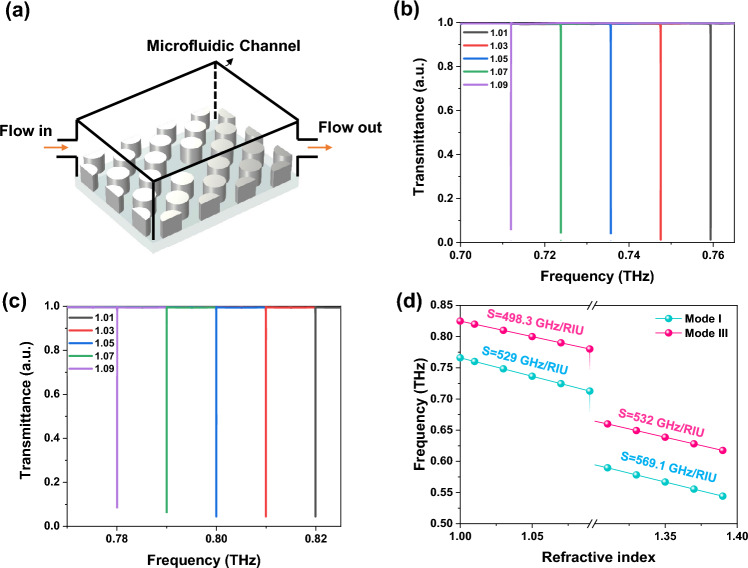
(**a**) Illustration of the suggested metasensor. Transmittance curves versus the refractive indices of gas (refractive index variations ranging from 1 to 1.1) (**b**) mode I, and (**c**) mode III. (**d**) Resonance frequencies versus the surrounding’s refractive index. A redshift in the q-BIC frequencies occurs when the refractive index of the sensing medium increases.

Finally, using the q-BIC for refractometric sensor applications, we define the bulk sensitivity, S = Δf/Δn, and figure of merit, FoM = S/FWHM to assess the sensing performance, where Δn is the analyte refractive index effect on the sensing environment, Δf represents the resonance frequency shift, and FWHM is the full width at half maximum of the resonance linewidth^49–54^. The suggested metasensor is shown in Fig. 10a. Regarding the fact that mode II is non-BIC, modes I and III are utilized for sensing applications. We take into account refractive index variations ranging from 1 to 1.1 for gases and 1.3 to 1.4 for covering the majority of aqueous and alcoholic liquids. The results in Fig. 10b and c indicate that a redshift in the BIC frequency occurs when the refractive index of the sensing medium increases, and has an insignificant impact on the modulation depth, and Q-factor. The redshift is explained by perturbation theory as $\left( {\delta \omega /\omega_{0} } \right) = - \left( {\iiint {\Delta \varepsilon \left| E \right|}^{2} dV/2\iiint {\varepsilon_{0} \left| E \right|}^{2} dV} \right)$, where $\delta \omega$, $\omega_{0}$, and $\Delta \varepsilon$ are resonance shift, resonance frequency, and change in the permittivity, respectively. Increasing the dielectric permittivity of the sensing environment leads to the redshift of the resonances^47^. As shown in Fig. 10d the sensitivities for liquid and gas-environment are S_l_ = 569.1 GHz/RIU and S_g_ = 529 GHz/RIU for mode I, and S_l_ = 532 GHz/RIU and S_g_ = 498.3 GHz/RIU for mode III, respectively. Additionally, the FoM values calculated for the suggested sensor are orders of magnitude greater than the traditional plasmonic sensors, with FoM_l_ = 669,412 RIU^−1^ and FoM_g_ = 623,176 RIU^−1^ for mode I, and FoM_l_ = 5,320,000 RIU^−1^ and FoM_g_ = 4,983,000 RIU^−1^ for mode III for liquid and gas, respectively. Assuming the sensor based on the proposed metasurface is characterized by a standard terahertz time-domain spectroscopy (THz-TDS) with a typical spectral resolution of 1 GHz, the limit of detection (LoD) for gas detection in mode I is 1.75 × 10^−3^ RIU, while for liquid detection it is 1.89 × 10^−3^ RIU. Additionally, in mode III, the LoD is 1.87 × 10^−3^ RIU for gas detection and 2.00 × 10^−3^ RIU for liquid detection. Furthermore, the proposed structure is a perfect candidate for sensing applications especially biosensing, because there are no ohmic losses that cause thermal heating and analyte degradation.

**Figure 11:**
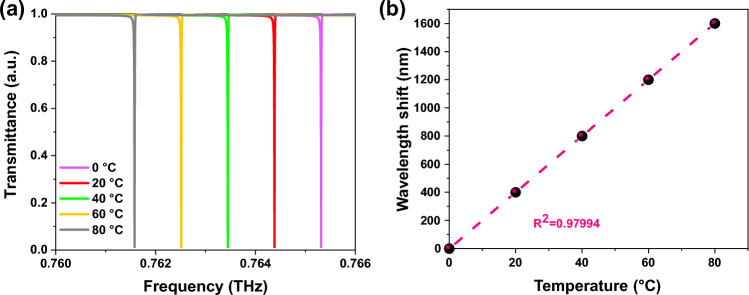
(**a**) Transmittance curves of mode I for various T. (**b**) Wavelength shifts of mode I with various T. As the temperature rises, the resonance of mode I displays a considerable redshift.

In practical sensing applications, the refractive index also depends on temperature (T)^55^. It is important to investigate the electromagnetic sensitivity of the structure at various temperatures. In this regard, the material thermal expansion and thermo-optical indices are crucial variables. The refractive index dependence on T is described as $n(T) = n(T_{0} ) + \alpha (T - T_{0} ) + \beta (T - T_{0} )$, where *T*_*0*_ is the room temperature, $\alpha$ and $\beta$ are the thermo-optic and thermal expansion coefficients, respectively. The thermo-optic and thermal expansion coefficients of Si are $1.88 \times 10^{ - 4} /K$ and $2.61 \times 10^{ - 6} /K$, respectively, and, the thermo-optic and thermal expansion coefficients of SiO_2_ are $8.6 \times 10^{ - 6} /K$ and $0.55 \times 10^{ - 6} /K$, respectively^56,57^. In our study, the water is used as a liquid sensing analyte, and the system is heated from 0 and 80 °C. The thermo-optic coefficient of pure water is $- 1.02 \times 10^{ - 4} /K$, so the refractive index of water diminishes as T rises^58^. The transmittance curves of mode I at various temperatures are displayed in Fig. 11a. It is apparent that as the temperature rises, the resonance of mode I displays a considerable redshift. The wavelength position change versus temperature is fitted linearly in Fig. 11b, and the temperature sensitivity can reach up to $S(T) = \Delta \lambda /\Delta T = 20.24nm/^\circ C$, which is higher than the results presented in Refs.^59^^,^^60^. As a result, the suggested metasensor can detect both the material temperature and refractive index. It should be noted that regarding the results already reported in Ref.^61^, the changes in the real and imaginary parts of the silicon refractive index caused by thermal carriers are much smaller than those caused by the thermo-optical effect presented here. Thus, there will be no significant shifts or broadening caused by thermal carriers. In addition, the sensitivity of the proposed structure to temperature changes makes it possible to tune the resonance frequencies without changing the structure dimensions and fabricating another device, as well as without using additional materials.

For the comparison between this research and earlier THz sensors, we have summarized the sensing results in Table 1. The results show that the proposed metasurface has higher sensitivity than the others except for Ref.^62^. However, the FoM for this particular structure is significantly lower compared to our proposed design. To assess the sensing capabilities of structures, the FoM provides a more comprehensive evaluation since it indicates both the sensitivity and Q-factor simultaneously. The high Q-factor enables precise monitoring of small spectral shifts at low analyte concentrations, increasing the accuracy of the detection process. Therefore, the ultra-high Q value of the bound state in the continuum modes gives the proposed sensor superior sensing capability compared to the previous works. It should be noted that the Q-factor and sensitivity are not the only parameters that comprehensive assessment of a sensor. Several other crucial factors can be considered to determine the practicality of a sensor. These factors include the limit of detection, which indicates the smallest quantity of analyte the sensor can detect; the sample-to-results duration, which represents the time it takes for the sensor to provide a measurement or outcome; the repeatability, which demonstrates the sensor's ability to consistently produce accurate results; and the selectivity, which measures the sensor's ability to specifically detect the target analyte without interference from other substances.

**Table 1:** Performance evaluations of earlier THz papers and the present work.

Ref	Material	Sensitivity (GHz/RIU)	Q-factor	FoM
^63^ (2020)	Silicon	77	140	11.1
^64^ (2021)	Aluminum	124.3	8.78	1.985
^43^ (2021)	LiTaO_3_	489	1.2 $\times$ 10^5^	25,352
^65^ (2019)	Silicon	226	270	64.7
^62^ (2022)	Gold	2500	777	547
^66^ (2019)	LiTaO3	438	3189	515
This work (MTD)	Silicon	569.1 for liquid and 529 for gas	9 $\times$ 10^5^	6.6 $\times$ 10^5^ for liquid and 6.2 × 10^5^ for gas
This work (ETD)	Silicon	532 for liquid and 498.3 for gas	5.1 $\times$ 10^6^	5.3 × 10^6^ for liquid and 4.9 × 10^6^ for gas

## Conclusion

To summarize, this work proposes a novel all-dielectric metastructure with three Fano modes. Modes I, II, and III electromagnetic sources are MTD, MTQ, and ETD, respectively. The sp-BIC drives modes I and III, which collapse into q-BIC resonances with an ultra-high-Q-factor of $9 \times 10^{5}$ and $5.1 \times 10^{6}$ at *α* = 2 μm, while mode II is induced by symmetric structures. A high-sensitive refractive index sensor with a sensitivity of 532 GHz/RIU for liquid detection and 498.3 GHz/RIU for gas detection that corresponds to an FoM of 5.3 × 10^6^ RIU^−1^, and 4.9 × 10^6^ RIU^−1^, respectively, is provided by the suggested structure. The materials used to design the metasurface have considerable thermo-optical and thermal expansion coefficients, enabling the temperature sensing capabilities of the proposed metasurface to be as sensitive as 20.24 nm/°C. The suggested metasurface is anticipated to obtain various applications including THz switches, multi-channel biochemical sensors, THz modulators, and nonlinear effects taking into account the features of multi-wavelength resonance, ultra-high Q value, significant spectral contrast, and impressive figure of merit.

### Supplementary Information

Supplementary Information.

## Data Availability

The datasets used and/or analyzed during the current study are available from the corresponding author upon reasonable request.
