# Supplementary material for: Quasi-BIC based all-dielectric metasurfaces for ultra-sensitive refractive index and temperature sensing
Source: Sci Rep. 2023 Nov 23;13:20625. doi: 10.1038/s41598-023-48051-2 (PMC10667344; doi:10.1038/s41598-023-48051-2)
Supplement: Supplementary file 1 — Supplementary Information. [file 41598_2023_48051_MOESM1_ESM.docx]

**Supplementary information**

**Quasi-BIC Based All-Dielectric Metasurfaces for Ultra-Sensitive Refractive Index and Temperature Sensing**

**Seyedeh Bita Saadatmand,^1^ Vahid ahmadi,^1,*^ and Seyedeh Mehri Hamidi,^2^**

^1^Faculty of Electrical and Computer Engineering, Tarbiat Modares University, Tehran, Iran.

^2^Magneto-plasmonic Lab, Laser and Plasma Research Institute, Shahid Beheshti University, Tehran, Iran.

* Corresponding author: [v_ahmadi@modares.ac.ir](mailto:v_ahmadi@modares.ac.ir)

**S1. Simulation settings**

We calculate the transmittance spectra, the near-field distribution, and the multimode decomposition calculation of the proposed metasurface by COMSOL Multiphysics (RF module). BIC frequencies are obtained from an eigenfrequency study in the RF module, and transmittance spectrum curves and resonance near-fields are determined through a frequency domain study. Fig. S1 shows that Floquet periodic boundary conditions are applied in the x and y directions. Additionally, two perfectly-matched layers (PML) are positioned at a specified distance from the structure along the z-axis to eliminate the reflection from the boundaries. To simulate transmittance spectra, a periodic port at the top is utilized to introduce incident waves. We employ an extremely fine-size physics-controlled mesh, utilizing free tetrahedral mesh for the physical domain and swept mesh for the PML domain.

Fig. S1. Schematics of the simulation settings

**S2. The feasibility of experimental fabrication**

Regarding the feasibility of experimental fabrication, the overall process for fabricating the proposed structure is presented in Fig. S2. Firstly, the SiO_2_ film can be deposited on the Si/SiO_2_ stack by the plasma-enhanced chemical vapor deposition (PECVD) technique. Secondly, the SiO_2_ film is spin-coated with the AZ5214E photoresist and baked. A photoresist is patterned by conventional UV photolithography. Next, the SiO_2_ layer is removed using dry etching with a mixture of CHF_3_ and CF_4_ gases and the remaining pattern is maintained as a protective mask for subsequent etching. Deep reactive-ion etching (DRIE) is employed to etch the silicon layer with the Bosch process. Finally, the photoresist and oxide layer are removed, and the structure is cleaned with deionized water.

Fig. S2. The suggested method for fabricating the proposed metastructure

**S3. Fabrication non-idealities**

To assess how the fabrication process tolerance affects the Q-factors and resonance frequencies of the proposed structure's q-BICs, we analyze the behavior of q-BICs when the structure parameters are changed by ± 5%. The results, as presented in Table S1, indicate that even with a ± 5% change in the metasurface parameters, the Q-factors of the q-BICs remain within the same order. Therefore, in practice, it can be expected that high Q-factors will be observed.

**Table S1- The effect of fabrication tolerance on q-BIC frequencies and Q-factors**

| Fabrication tolerance | | f_res_ (P/h/r) THz | f_res_  (P/h/r-5%)  THz | f_res_ (P/h/r+5%)  THz | Q-factor (P/h/r) | Q-factor (P/h/r-5%) | Q-factor (P/h/r+5%) |
| --- | --- | --- | --- | --- | --- | --- | --- |
| P_x_, P_y_$\text{±}$5% | mode I | 0.7660238 | 0.7670236 | 0.7650245 | 9$\text{×}$10^5^ | 8.98$\text{×}$10^5^ | 9.04$\text{×}$10^5^ |
|  | mode III | 0.8248923 | 0.8258909 | 0.8238941 | 5.1$\text{×}$10^6^ | 5.09$\text{×}$10^6^ | 5.1$\text{×}$10^6^ |
| h$\text{±}$5% | mode I | 0.7660238 | 0.7740238 | 0.7582238 | 9$\text{×}$10^5^ | 9$\text{×}$10^5^ | 9$\text{×}$10^4^ |
|  | mode III | 0.8248923 | 0.8268923 | 0.82289931 | 5.1$\text{×}$10^6^ | 5.11$\text{×}$10^5^ | 5.07$\text{×}$10^5^ |
| r$\text{±}$5% | mode I | 0.7660238 | 0.7740238 | 0.7565438 | 9$\text{×}$10^5^ | 8.97$\text{×}$10^5^ | $\text{9}\text{×}$10^4^ |
|  | mode III | 0.8248923 | 0.8358923 | 0.8123923 | 5.1$\text{×}$10^6^ | 5$\text{×}$10^6^ | 5.23$\text{×}$10^6^ |
